# A Meta-Analysis of Line Bisection and Landmark Task Performance in Older Adults

**DOI:** 10.1007/s11065-021-09505-4

**Published:** 2021-04-22

**Authors:** Gemma Learmonth, Marietta Papadatou-Pastou

**Affiliations:** 1 Institute of Neuroscience & Psychology, University of Glasgow, Glasgow, Scotland; 2 School of Education, National and Kapodistrian University of Athens, Athens, Greece; 3 Biomedical Research Foundation, Academy of Athens, Athens, Greece

**Keywords:** Spatial attention, Pseudoneglect, Aging, Line bisection, Landmark task, HAROLD model

## Abstract

Young adults exhibit a small asymmetry of visuospatial attention that favours the left side of space relative to the right (*pseudoneglect*). However, it remains unclear whether this leftward bias is maintained, eliminated or shifted rightward in older age. Here we present two meta-analyses that aimed to identify whether adults aged ≥50 years old display a group-level spatial attention bias, as indexed by the *line bisection* and the *landmark* tasks. A total of 69 datasets from 65 studies, involving 1654 participants, were analysed. In the meta-analysis of the line bisection task (*n* = 63), no bias was identified for studies where the *mean age* was ≥50, but there was a clear leftward bias in a subset where all *individual participants* were aged ≥50. There was no moderating effect of the participant’s age or sex, line length, line position, nor the presence of left or right cues. There was a small publication bias in favour of reporting rightward biases. Of note, biases were slightly more leftward in studies where participants had been recruited as part of a stand-alone older group, compared to studies where participants were recruited as controls for a clinical study. Similarly, no spatial bias was observed in the meta-analysis of the landmark task, although the number of studies included was small (*n* = 6). Overall, these results indicate that over 50s maintain a group-level leftward bias on the line bisection task, but more studies are needed to determine whether this bias can be modulated by stimulus- or state-dependent factors.

## Introduction

Healthy young adults exhibit a consistent, group-level, lateralised asymmetry of spatial attention favouring the left side of space (“*pseudoneglect*”, Bowers & Heilman, 1980). This phenomenon typically results in an overestimation of features located within the left hemispace relative to the right, such as the size (Nicholls et al., 1999), number (Luh, 1995), and the luminance of objects (Mattingley et al., 1994) as well as their spatial frequency (Niemeier et al., 2007). In contrast, many studies in older adults (usually aged 50-60 years upwards) report a different pattern of spatial attention, with group-level lateralised biases either eliminated entirely (Failla et al., 2003; Learmonth et al., 2017) or mirrored, favouring the right hemispace in a pattern similar to that observed in hemispatial neglect (Benwell et al., 2014; Fujii et al., 1995; Fukatsu et al., 1990; Schmitz & Peigneux, 2011; Stam & Bakker, 1990). However, other studies have reported a maintained leftward pseudoneglect bias in this older group (Beste et al., 2006; Brooks et al., 2016; Hatin et al., 2012; Varnava & Halligan, 2007). A meta-analysis of pseudoneglect performed 20 years ago by Jewell and McCourt (2000) identified that spatial biases were indeed more rightward in adults aged over 50 years old compared to those aged under 40. Yet, there remains considerable debate regarding whether spatial biases are maintained leftward or shifted rightward in the older population or whether such a bias exists at all (Friedrich et al., 2018).

Our understanding of these possible age-related changes in behavioural measures of spatial bias is important because these measures are often used as a proxy measurement of lateralised neural activity within the brain. Specifically, pseudoneglect is considered to be a manifestation of right posterior parietal dominance for spatial attention, thus directing our attention to the contralateral left hemispace (Benwell et al., 2014; Çiçek et al., 2009; Fink et al., 2001; Foxe et al., 2003). As such, a rightward shift of spatial bias in older adults could potentially represent an age-related reorganisation or repurposing of the brain regions that are responsible for spatial processing. On the contrary, if older adults exhibit a maintained pseudoneglect, then this could indicate that spatial attention is not susceptible to the same aging processes that affect other cognitive functions. For example, empirical findings in the working memory domain have led to the development of various theoretical frameworks to account for observed patterns of cortical reorganisation in the elderly. By extending these frameworks to spatial processing, we may be led to conclude that an eliminated or mirrored spatial bias indicates that the right hemisphere undergoes a process of accelerated aging relative to the left (the *right hemi-aging* model; Goldstein & Shelly, 1981). Alternatively, eliminated spatial biases could represent hemispheric asymmetry reduction in older adults (the HAROLD model; Cabeza et al., 1997), where brain functions that are lateralised in young adults become less lateralised in older age. Later modifications of the HAROLD model proposed that contralateral neural resources (i.e., left-hemispheric in this case) may be specifically co-opted in order to support behavioural performance (Cabeza et al., 2002; Dolcos et al., 2002). In addition to deepening our knowledge of how the brain ages, given that altered lateralised spatial processing in the elderly has been linked to an increased risk of falls (Nagamatsu et al., 2009, 2011, 2013), our understanding of these group-level changes of spatial bias in seniors could potentially represent a simple method of identifying at-risk individuals. Moreover, identifying any age-related changes in neural activation should allow for a more targeted approach to the delivery of non-invasive brain stimulation and neurofeedback therapies (Learmonth et al., 2015b).

Although there appears to be some potential in linking spatial biases to lateralised neural activity at an individual level, and further to ‘real-world’ behaviours, it is important to emphasise that the bulk of research in this area describes spatial biases at the group level, with few studies focusing on individual differences (although see Benwell et al., 2013; McCourt, 2001; Newman et al., 2014; Szczepanski & Kastner, 2013). It is also important to highlight that the spatial biases that are observed in individual healthy adults are subtle, often representing only a few millimetres of deviation from the veridical midpoint of a bisected line. This is particularly apparent in comparison to the large right-sided biases observed in patients with hemispatial neglect (Halligan et al., 1990). Large variations in the range of biases are typically observed within age groups, both in terms of bias direction and magnitude, and there is often considerable overlap when comparing young and older adults directly (see Friedrich et al., 2016; Learmonth et al., 2018). Thus, some older adults exhibit a leftward bias that is similar to, or indeed even larger, than young adults. Conversely, some young adults exhibit a large rightward bias that is consistent across testing days (Learmonth et al., 2015a). As such, it is important not to over-interpret small group-level changes of bias in terms of possible cortical reorganisation of spatial attention in older adults, when the conclusions may not be applicable to all, or even the majority of individuals within the group.

One potential source of the heterogeneity described above is the range of tasks that are administered to quantify spatial biases (Failla et al., 2003; Friedrich et al., 2018). The line bisection task, which involves participants indicating the midpoint of a horizontally presented line, is most prevalent across the literature. Given the simplicity of the line bisection task, it is often used at the hospital bedside in the diagnosis of hemispatial neglect (Schenkenberg et al., 1980). The landmark task is a non-motor adaptation of the line bisection task, where participants indicate whether the left or right side of a pre-bisected line is shorter or alternatively whether the bisection mark is closer to the left or right end of the line (Milner et al., 1992). Other tasks are also used to assess spatial biases, including the greyscales task (Mattingley et al., 1994), gratingscales task (Niemeier et al., 2007), chimeric faces (Levy et al., 1972), and lateralised visual detection (Learmonth et al., 2015a). However, we have shown in young adults that although individual tasks elicit consistent measures of spatial bias over time, the direction and magnitude of biases do not necessarily correlate across the different tasks (Learmonth et al., 2015a, 2018; Luh, 1995; Nicholls et al., 1999). We have also recently identified a similar pattern of good intra-, but poor inter-task consistency in older adults aged over 60 years old (Märker et al., 2019). Further compounding this heterogeneity, it is likely that subtle variations in the stimulus properties presented within each task place varying cognitive demands on participants, leading to stimulus-induced shifts of spatial bias, for example line length (Benwell et al., 2014; Mennemeier et al., 2001), the spatial position of the line (McCourt et al., 2000; McCourt & Jewell, 1999) and the presence of lateralised cues (McCourt et al., 2005; Mennemeier et al. 1997). Participant characteristics such as sex (Chen & Goedert, 2012; Pierce et al., 2003) and handedness (De Agostini et al., 1999; Failla et al., 2003), and endogenous states, such as transient alertness levels (Dufour et al., 2007; Manly et al., 2005) are also likely to influence spatial biases, perhaps even in an additive manner (Benwell et al., 2014; McCourt & Jewell, 1999).

In summary, it remains unclear whether older adults, as a group, exhibit maintained pseudoneglect, an eliminated spatial bias, or a preference for the right hemispace when tested with the line bisection and landmark tasks. Here, we report the results of an updated and enriched meta-analysis of the literature to date, given that a substantial number of studies have been conducted since the meta-analysis of Jewell and McCourt (2000). The principal question is whether older adults, aged ≥50 years old, have a spatial attention bias that is statistically different to zero (i.e., no bias to either side of space). Due to the range of tasks that are used to measure spatial biases, and the problematic nature of comparing the biases obtained across different tasks, we chose to focus only on the line bisection and the landmark tasks. Secondly, in order to maximise the number of datasets in the meta-analysis, we included data from healthy older adults who had been recruited as control samples in clinical studies. We also aimed to assess the moderating influence of age, sex, line position, the presence of lateralised cues, and the type of task presented. Finally, we determined whether small study bias, a form of publication bias, has affected the reporting of spatial biases in older adults within the wider literature.

## Method

The protocol for the meta-analysis was pre-registered on Open Science Framework prior to conducting the literature searches (https://osf.io/d97qc/).

Studies were identified from literature searches using the PsycINFO, Web of Science and Scopus electronic databases, from inception until 12^th^ January 2021. The search terms ("landmark task" OR "line bisection") AND (“aging” OR “ageing” OR “older”) were used, targeting all fields. After rejecting duplicate records, 1620 unique abstracts were screened for potential inclusion using the criteria listed in the “Study selection” section below. The full text of 211 articles was examined to assess for eligibility and 65 studies (comprising a total of 69 datasets) were identified as eligible for inclusion in the meta-analysis. The available data was extracted, cross-checked by a second reviewer and any discrepancies were resolved by consultation with a third reviewer.

**Fig. 1:**
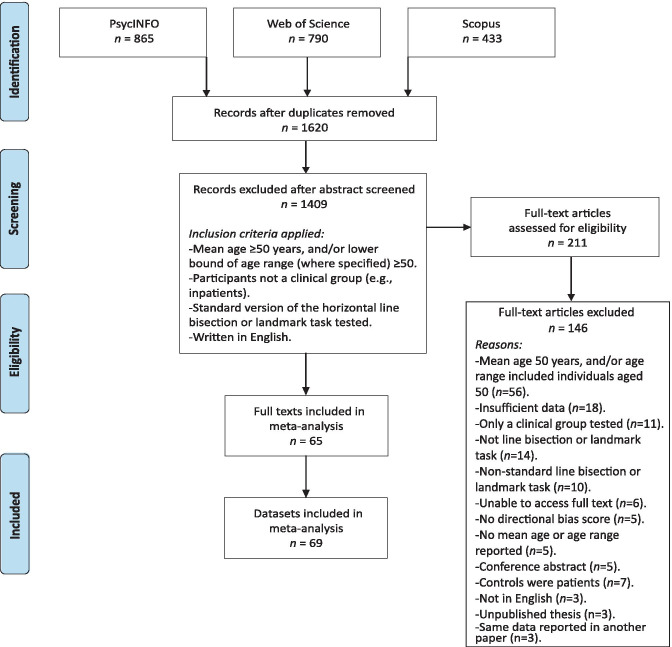
Flow diagram documenting the electronic database searches, the screening of study abstracts and full-texts, and the reasons for excluding studies. Adapted from Preferred Reporting Items for Systematic Reviews and Meta-Analyses: The PRISMA Statement (Moher et al., 2009)

Additional information was obtained by email correspondence for 8 studies where there was insufficient data reported to calculate effect sizes (Benwell et al., 2014; Beste et al., 2006; Ellis et al., 2006; Jeong et al., 2006; Laudate et al., 2013; Luauté et al., 2012; Vallar et al., 2000; Veronelli et al., 2014a). A further 18 studies may have been eligible for inclusion but the requested data was either not available, the authors failed to respond, or contact details were no longer valid (Andrade et al., 2013; Baheux et al., 2007; Bailey et al., 2000; Bourne & Gray, 2009; Costantini et al., 2014; Ebersbach et al., 1996; Gutiérrez Pérez et al., 2011; Harvey et al., 1995a; Kim et al., 2011; Lee et al., 2010, 2001; Luvizutto et al., 2020; Olk et al., 2004; Pizzamiglio et al., 1990, 2000; Wang et al., 2005; Reuter-Lorenz & Posner, 1990; Rousseaux et al., 2006). In contravention of our pre-registration we did not contact authors to ask for unpublished studies due to time constraints. Details of the search process are documented in Fig 1. The PRISMA statement (Moher et al., 2009) on reporting items for systematic reviews and meta-analyses was followed.

### Study Selection

The criteria for inclusion of a study in the meta-analysis were as follows:

1. **Age:** Studies were included if the participant sample consisted of adults aged ≥50 years old. Studies were excluded if the age range included any participants aged <50 years old. If no age range was reported, but the mean age was ≥50, the study was included.
2. **Clinical status: **Studies were included if the participants were healthy individuals. We also included studies if the participants formed a control group that had been recruited as a comparator to a clinical group, for example hemispatial neglect (e.g., Halligan et al., 1990) and dementia (e.g., Almkvist et al., 1992), but only the data on the healthy controls were used for the purposes of the meta-analysis. Studies where the control group were themselves part of a clinical population (e.g., hospital inpatients; Schenkenberg et al., 1980) were excluded.
3. **Task:** Studies were included where visuospatial bias was assessed using standard versions of either the line bisection task or the landmark task. Non-standard versions, such as gap bisection (e.g., Balconi et al., 2013), sentence bisection (Arduino et al., 2016), and rods that were bisected via tactile exploration (Brooks et al., 2011), were excluded. Only lines presented horizontally were included, and those presented vertically (e.g., Lee et al., 2002) or radially (e.g., Barrett & Craver-Lemley, 2008) were excluded.
4. **Language: **Only studies written in English were included.

### Moderators

The following moderator variables were examined in the meta-analysis:

1. **Age:** The numerical mean age of participants in each study was included as a continuous variable. All of the studies that were included reported the mean age, except three. The control participants in Grossi et al. (1999) were the “same age [] as the patients” (i.e., 50-69 years old). Similarly, Luauté et al. (2012) did not report a mean age for their control participants, but recruited “six healthy subjects and five patients aged between 67 and 80 years old”. Van Deusen (1983) was included although the author reported that 1 of the 93 participants *may* have been aged <50 (although this was considered unlikely). Finally, as an exploratory follow-up, an overall effect calculation was performed separately for studies where all participants were known to be aged at least 50 and for studies where the age range of participants was not reported and the two models were then compared.
2. **Sex:** Nine studies reported separate data for men and women (Barrett et al., 2002; Barrett & Craver-Lemley, 2008; Beste et al., 2006; De Agostini et al., 1999; Halligan et al., 1990; Learmonth et al., 2018; Muayqil et al., 2019; Pierce et al., 2003; Varnava & Halligan, 2007). Since the majority of studies did not report spatial biases separately for male and female participants, we also calculated the percentage of male participants in each study where this data was available. Nineteen studies did not report the sex of their participants and the data from the following studies were therefore not included in this analysis (Andrews et al., 2017; Barton et al., 1998; Binetti et al., 2011; Corazzini et al., 2005; Daini et al., 2008; Doricchi et al., 2002; Gassama et al., 2011; Grossi et al., 1999; Hatin et al., 2012; Luauté et al., 2012; Mennemeier et al., 1997, 2001; Plummer et al., 2006; Richard et al., 2004; Striemer & Danckert, 2010; Vallar et al., 2000; Veronelli et al., 2014b; Williamson et al., 2018; Zeller & Hullin, 2018).
3. **Hand used:** The hand that was used to bisect the line was experimentally tested in five studies, the left and right hands in Beste et al. (2006), De Agostini et al. (1999), Hatin et al. (2012) and Jeong et al. (2006), and the left, right, and both hands simultaneously in Failla et al. (2003).
4. **Line length:** Eleven studies reported spatial biases separately for more than one line length (Benwell et al., 2014; Binetti et al., 2011; Cowey et al., 1994; Ellis et al., 2006; Halligan et al., 1990; Kasai et al., 2016; Learmonth et al., 2017; Potter et al., 2000; Richard et al., 2004; Vallar et al. 2000; Varnava & Halligan, 2007). The visual angle of each line was extracted, or calculated manually from the line length and the viewing distance when the visual angle was not reported. As per Jewell and McCourt (2000) a viewing distance of 45cm was assumed where the viewing distance was unavailable. Eight studies reported data from three or more line lengths (Benwell et al., 2014; Binetti et al., 2011; Cowey et al., 1994; Ellis et al., 2006; Halligan et al., 1990; Richard et al., 2004; Vallar et al., 2000; Varnava & Halligan, 2007). To aid the comparison between long and short lines, we selected only the longest and shortest lines that were reported in these studies, rather than calculating an average across a range of lengths. This resulted in a mean *long* line length of 25.16° and *short* line length of 5.18°, which corresponds with the allocation of long lines as ≥17.68° and short lines as ≤16.44° in Jewell and McCourt (2000). A further 4 studies reported data from multiple line lengths but were not included in the line length analysis, as the lines were either all *short* or all *long* according to our criteria (Barrett et al., 2002; Barton et al., 1998; Drago et al., 2008a; Veronelli et al., 2014a).
5. **Spatial position:** The horizontal spatial position of the line was examined in 9 studies (Beste et al., 2006; Ellis et al., 2006; Jeong et al., 2006; Learmonth et al., 2018; Mennemeier et al., 1997, 2001 [experiments 1 and 2]; Potter et al., 2000; and Williamson et al., 2018). Data was extracted for lines that were positioned to the left and right of the participant’s midline.
6. **Cues:** Seven studies presented lateralised cues that aimed to direct attention to the left or right hemispace during the line bisection task. This primarily involved the presentation of a stimulus located at the left or right ends of the line (a letter: Harvey et al., 1995b; 2000; a letter or number: Mennemeier et al., 1997; dots: Drago et al., 2008a, 2008b; circles: Williamson et al., 2018), or where a section of the line was thicker at one end (Falchook et al., 2015). Three additional studies reported data for cued bisections but were excluded, one where no means or standard deviations were available (Chieffi et al. 2014), one which involved a horizontally moving background (Choi et al., 2007), and another which involved Müller-Lyer illusion lines (Olk et al., 2001).
7. **Control group status:** Finally, we assessed whether the participant recruitment context had an effect on spatial bias. Specifically, whether biases differed in participants who were recruited as control participants within a clinical study, compared to those recruited as a stand-alone healthy older participant group. Forty-two datasets that were included in the analysis had tested an older control group as part of a clinical study, and 22 datasets had tested a stand-alone group of healthy older adults.

In addition to these 7 moderator variables, as per our pre-registered protocol we also extracted data for the handedness of participants, the modality of presentation (paper and pencil or computerised), the salience of the line, eye of regard (binocular or uniocular), gaze direction, scanning direction, and body orientation. We either did not find enough data to perform analyses on these moderator variables or in the case of handedness the majority of studies had exclusively recruited right-handed participants (44 of the 49 studies that reported participant handedness). The raw data for these moderators and analysis code is available on Open Science Framework at https://osf.io/rme53/.

### Effect Size Estimates

As per convention, leftward biases were denoted by a negative value and rightward biases with a positive value. The mean bias scores and standard deviations were converted to effect size estimates using the formula:

$$Cohe{n}^{\prime}s \ d=mean/SD$$

In cases where studies reported the mean bias and standard deviations for multiple experimental factors (e.g., sex, line lengths) rather than an overall mean bias, the effect sizes were calculated separately for each factor and subsequently averaged to create an aggregate effect size. In a few cases (Daini et al., 2008; Harvey et al., 2000, 1995b; Schmitz & Peigneux, 2011; Veronelli et al., 2014b; Williamson et al., 2019), only the *t*-value obtained from a one-sample *t*-test against chance was reported and this was used to calculate Cohen’s *d* using the formula:

$$Cohe{n}^{^{\prime}}s \ d=t/sqrt(n)$$

In other cases, the standard deviation was calculated first from reported confidence intervals (Andrews et al., 2017) or standard errors (Plummer et al., 2006; Striemer & Danckert, 2010; Williamson et al., 2018) before proceeding to the effect size calculation. The rationale for the decisions taken in each study are reported in Table 1.

### Statistical Analysis

The *metafor* (Viechtbauer, 2007) and *robumeta* (Fisher et al., 2017) software packages for R were used for the analysis. A single, overall analysis was not possible, due to the fact that two different tasks were used in the datasets (line bisection and landmark) in quite unequal numbers (63 and 6, respectively). Moreover, two of the datasets reported data on the same participants for the two tasks (Harvey et al., 2000; Learmonth et al., 2018), violating the independence of data points. Therefore, the two tasks were analysed separately and their overall effect sizes were then compared.

First, for each of the datasets, the effect size was calculated as described above. Then, for each of the two analyses an overall effect size estimate was calculated by weighting each dataset effect size according to sample size (an index of study precision), using a random-effects model along with a test for the overall effect (*Z* statistic with corresponding *p*-value). Heterogeneity was then tested using three tests: the *Q* statistic (with its corresponding *p*-value), the *I*^*2*^ index, and the Tau^2^ statistic. The *I*^*2*^ index levels can be described as low, moderate, and high, when they fall close to 25%, 50%, and 75% respectively (Higgins et al., 2003). The overall effect sizes calculated for the two tasks were then compared using the *Q* statistic (with corresponding *p*-value).

In order to investigate the presence of publication bias we used the funnel plot graphical test, Egger’s *t* statistical test, and Duval and Tweedie's (2000) trim and fill method of correcting bias. A moderator variables analysis was further deemed necessary, as the heterogeneity found may be caused by the presence of moderator variables. This analysis was performed only within the line bisection group of studies, that included a sufficient number of datasets to justify such an analysis. For continuous moderator variables (i.e., age and percentage of male participants) meta-regression was used, using a random effects model (method of moments), with evaluation in terms of the *Q* statistic, *R*^*2*^ (and *p*-value). When examining the effects of the categorical moderator variables (i.e., sex, line length, presence of lateralised cues, line position, and control group status) the average effect sizes in the different subgroups that form the levels of the moderator were compared again by means of the *Q* statistic (and *p*-value). Forest plots and funnel plots were used to depict the information visually.

## Results

A total of *k*_*t*_ = 65 studies were included in the two meta-analyses, comprising *k*_*d*_ = 69 separate datasets and totalling *n*_*t*_ = 1654 individuals. Details of individual studies and moderator variables are shown in Tables 1 and 2.

**Table 1:** Study characteristics of studies using the line bisection task

	Study	Mean Age	N	Task	Recruited as a clinical control group?	Effect size (Cohen’s *d*)	Moderator analysis	Additional information
1	Almkvist et al. (1992)	79.3	18	LB	Y (dementia)	-0.26	A, ER, S_2_	Only subjects without white matter intensities included. Mean and SD provided separately for right eye and left eye presentations. *d* calculated separately for each, then averaged.
2	Andrews et al. (2017)	69.3	12	LB	N	-0.26	A, H	Mean and 95% CI reported. SD calculated from CIs using RevMan 5.3 software. *N* = 2 excluded from analysis.
3	Barrett et al. (2002)	65.6	30	LB	N	-0.31	A, H, S_1_, S_2_	-
4	Barrett and Craver-Lemley (2008)	73.7	60	LB	N	-0.18	A, H, S_1_, S_2_	-
5	Barton et al. (1998)	59.2	9	LB	Y (neglect/ hemianopia)	1.15	A	Mean and SD provided for long and short lines. *d* calculated separately for each, then averaged.
6	Beste et al. (2006)	60.5	140	LB	N	-0.24	A, H, HU, S_1_, S_2_, SP	Raw data obtained from authors. *d* calculated separately for males and females by averaging right and left hand used data for each. Overall *d* calculated by averaging males and females.
7	Binetti et al. (2011)	67.9	50	LB	N	0.2	A, LL	*Closed empty* condition extracted. Mean and SD reported separately for 4 line lengths. *d* calculated separately for each, then averaged. Long (160mm = 20.16°) and short (20mm = 2.55°) lines used in line length analysis.
8	Bisiach et al. (1998)	64.6	40	LB	Y (neglect)	0.32	A, H, S_2_	Mean reported but no SD. 3SD above mean reported as 10.34. Calculated 1SD using formula (3SD-mean)/3.
9	Brooks et al. (2016)	69.8	59	LB	N	-0.49	A, H, S_2_, SD	*N* = 1 excluded from analysis.
10	Chiba et al. (2006)	63.9	21	LB	Y (neglect)	0.03	A ,H, S_2_, SD	Mean and SD reported for 2 scanning directions (left to right and right to left). *d* calculated separately for each, then averaged.
11	Chieffi et al. (2014)	69.0	20	LB	N	0.003	A, H, S_2_	-
12	Choi et al. (2007)	66.4	22	LB	Y (neglect)	-0.19	A ,H, S_2_	Mean and SD reported for large and small backgrounds. *d* calculated separately for each, then averaged.
13	Corazzini et al. (2005)	70.7	10	LB	Y (neglect)	0.5	A, H	-
14	Cowey et al. (1994)	73.0	2	LB	Y (neglect)	-0.20	A, LL, S_2_	Raw data reported for 4 conditions (near space, far space pointer, near space pointer and far space pointer repeated). Long (305mm near/166cm far space = 37.5°) and short (51mm near/21cm far space = 6.33°) lines for each condition were averaged and used in the line length analysis.
15	Cowey et al. (1998)	76.0	2	LB	Y (neglect)	-0.88	A, S_2_	Raw data reported for 6 length/distance conditions.
16	Daini et al. (2008)	71.4	12	LB	Y (cerebellar damage)	-1.04	A, H	T-statistic vs 0 reported and used to calculate *d*.
17	De Agostini et al. (1999)	74.6	66	LB	N	-0.48	A, H, HU, S_1_, S_2_	Mean and SD reported for 4 groups (men and women, using right and left hands). *d* calculated separately for each, then averaged.
18	Doricchi et al. (2002)	65.9	10	LB	Y (neglect)	0.16	A	LM also tested but no data reported.
19	Drago et al. (2008a)	68.1	10	LB	Y (Alzheimer’s)	0.17	A, C, S_2_	Mean and SD reported for 3 line lengths. *d* calculated separately for each, then averaged. Left dot and right dot conditions extracted for the cue analysis.
20	Drago et al. (2008b)	69.6	10	LB	Y (Parkinson’s)	0.04	A, C, S_2_	Data extracted for the *Combined No Dots* condition for the overall bias score. *Combined Right dots* and *Combined Left dots* extracted for the right and left cue conditions.
21	Ellis et al. (2006)	76.6	20	LB	Y (neglect)	-0.73	A, LL, S_2_, SP	Raw data obtained from authors. Longest (160mm = 29.86°) and shortest (20mm = 3.82°) lines used in line length analysis. Left and right lines used in spatial position analysis.
22	Failla et al. (2003)	66.1	30	LB	N	-0.38	A, H, HU, S_2_	-
23	Falchook et al. (2015)	70.6	9	LB	Y (Parkinson’s)	-0.24	A, C, H, S_2_	Mean and SD reported for the 3 standard line types (0.06, 0.2 and 2.5cm thick). *d* calculated separately for each, then averaged. Left and right cue conditions were calculated from the 4 lines where the left or right segment of the line respectively was thicker. *d* calculated for each, then averaged.
24	Fujii et al. (1995)	70.1	36	LB	N	0.29	A, H, S_2_	-
25	Gassama et al. (2011)	64.5	12	LB	Y (neglect)	-0.61	A, H, SA	Data extracted from the sitting condition.
26	Goedert et al. (2010)	72.8	12	LB	N	0.14	A, H, S_2_, SA	-
27	Göttler et al. (2018)	70.1	24	LB	Y (carotid stenosis)	1.6	A, H, S_2_	-
28	Grossi et al. (1999)	-	5	LB	Y (neglect)	0.92	-	-
29	Halligan et al. (1990)	69.3	20	LB	Y (neglect)	0.13	A, H, LL, S_1_, S_2_	Raw data reported and used to calculate mean and SD for males, females and overall. Shortest (25mm = 3.18°) and longest (279mm = 34.45°) lines used in line length analysis.
30	Harvey et al. (1995b)	66.2	12	LB	Y (neglect)	-0.68	A, H, S_2_	T-statistic vs 0 reported and used to calculate *d*. LM also tested but no data reported.
31	Harvey et al. (2000)	71.0	18	LB	N	0.12	A, C, H, S_2_	T-statistic vs 0 reported for *left cue* and *right cue* conditions. *d* calculated separately for each, then averaged.
32	Harvey et al. (2002)	70.8	13	LB	Y (neglect)	-0.4	A, H, S_2_	-
33	Hatin et al. (2012)	72.7	12	LB	N	-0.17	A, H, HU	-
34	Jeong et al. (2006)	70.5	6	LB	Y (hydrocephalus)	-0.2	A, H, HU, S_2_, SP	Mean and SD obtained from authors for 6 conditions (left and right hand used; left, right and centred line position). *d* calculated separately for each then averaged.
35	Kasai et al. (2016)	81.0	40	LB	Y (Alzheimer’s)	-0.02	A, LL, S_2_	Mean and SD reported for long (200mm = 25.06°) and short (120mm = 15.19°) lines. *d* calculated separately then averaged.
36	Laudate et al. (2013)	70.0	11	LB	Y (Parkinson’s)	0.39	A, H, S_2_	Raw data for centred lines obtained from authors.
37	Learmonth et al. (2018)	70.4	39	LB	N	-0.27	A, H, S_1_, S_2_, SP	Raw data obtained from authors.
38	Lee et al. (2004)	69.7	40	LB	N	-0.11	A, H, S_2_	Mean and SD extracted for the *solid line bisection* condition.
39	Liu et al. (2004)	73.9	21	LB	Y (Alzheimer’s)	-0.36	A, H, S_2_	-
40	Luauté et al. (2012)	-	6	LB	Y (neglect)	-0.07	-	Raw data obtained from authors. Data extracted for *baseline* condition.
41	Mańkowska et al. (2019)	70.3	23	LB	N	-0.37	A, H, S_2_	-
42	Mennemeier et al. (1997)	71.9	10	LB	Y (left or right hemisphere lesion)	0.6	A, C, H, SA, SP	Mean and SD extracted for the signed errors in 3 conditions (*No cue Left hemispace*, *No cue Right hemispace* and No cue *Center space*). *d* calculated separately for each, then averaged. Data for the cue analysis obtained by calculating *d* separately for left, right and centrally presented lines with left cues then averaging, and the same procedure for right cues.
43	Mennemeier et al. (2001)	72.0	11	LB	Y (neglect)	0.96	A, H, SP	Exp 1. Mean and SD reported for left, right and centrally positioned lines. *d* calculated separately, then averaged.
44	Mennemeier et al. (2001)	72.0	11	LB	Y (neglect)	-1.21	A, H, SP	Exp 2. Mean and SD reported for left, right and centrally positioned lines. *d* calculated separately, then averaged.
45	Muayqil et al. (2019)	58.69	18	LB	N	0.69	A, S_1,_ S_2_	Mean and SD reported for males and females separately and for ages 50-59 and >60. *d* calculated separately, then averaged.
46	Olk et al. (2001)	71.7	15	LB	Y (neglect)	-0.33	A, H, S_2_	Mean and SD reported for baseline condition (non-Müller-Lyer lines).
47	Pierce et al. (2003)	71.3	30	LB	N	-0.06	A, H, S_1_, S_2_	Mean and SD reported for men and women at Time 1 and Time 2. *d* calculated separately for each, then averaged.
48	Plummer et al. (2006)	70.3	10	LB	Y (neglect)	0.33	A, H, SD	Mean and standard errors reported. SE converted to SD using formula SD=SE*√N. The *No Stimulus mean* condition was extracted for the overall bias. Data for the scanning direction analysis was obtained from the *No stimulus Left start*, and *No stimulus Right start* conditions.
49	Potter et al. (2000)	72.8	13	LB	Y (neglect)	0.21	A, LL, S_2_, SP	Mean and SD reported for 8 conditions: left top, right top, left bottom, right bottom line positions for short (50mm = 6.36°) and long (140mm = 17.68°) lines. *d* calculated separately, then averaged.
50	Richard et al. (2004)	54.0	8	LB	Y (neglect)	0.9	A, H, LL	Mean and SD reported for 4 line lengths. *d* calculated separately, then averaged. Shortest (25mm = 3.18°) and longest (200mm = 25.06°) lines used in line length analysis.
51	Salazar et al. (2019)	62.9	67	LB	Y (Parkinson’s)	0.27	A, S_2_	-
52	Sposito et al. (2010)	66.0	15	LB	Y (neglect)	0.17	A, S_2_	-
53	Striemer and Danckert (2010)	67.0	8	LB	Y (neglect)	-0.07	A, H	Mean and SD reported pre-prism adaptation. LM also tested but baseline mean and SD not reported.
54	Ulm et al. (2013)	68.0	10	LB	Y (neglect)	-0.5	A,H, S_2_	-
55	Vallar et al. (2000)	62.7	6	LB	Y (neglect)	0.2	A, H, LL	Mean and SD obtained from authors (baseline condition *without fins*) for 3 line lengths. *d* calculated separately for each, then averaged. Shortest (80mm = 10.16°) and longest (240mm = 29.86°) lines used in line length analysis.
56	Van Deusen (1983)	75.0	93	LB	N	-0.14	S_2_	Mean and SD reported for *left positioned plus centrally positioned lines* (right-positioned lines were not reported). n.b. One of the 93 subjects may have been aged <50.
57	van Dijck et al. (2012)	68.6	12	LB	Y (neglect)	0.52	A, H	Mean regression intercept and SD across 3 line lengths reported.
58	Varnava and Halligan (2007)	65.7	60	LB	N	-0.4	A, H, LL, S_1_, S_2_	Mean and SD reported for 18 conditions (3 line lengths, 3 age groups, 2 genders). *d* calculated separately for each, then averaged. Longest (180mm = 22.62°) and shortest (20mm = 2.55°) lines used for line length analysis.
59	Veronelli et al. (2014a)	68.7	11	LB	Y (neglect)	0.07	A, S_2_	Mean and SD obtained from authors for *long* and *short* lines. Not used in line length analysis because both <17° (short).
60	Veronelli et al. (2014b)	77.9	8	LB	Y (neglect)	0.69	A, S_2_	T-statistic vs 0 reported and used to calculate *d*.
61	Veronelli et al. (2014b)	74.7	6	LB	Y (neglect)	0.61	A	T-statistic vs 0 reported and used to calculate *d*.
62	Williamson et al. (2018)	57.0	8	LB	Y (neglect)	-0.16	A, C, H, SP	Least squares means of fixed effects and standard errors reported for left, right and centrally positioned lines. SE converted to SD using formula SD=SE*√N. *d* calculated separately, then averaged. *Left* and *right distractor* conditions used for cue analysis.
63	Williamson et al. (2019)	68.0	37	LB	N	-0.253	A, H, S_2_	T-statistic vs 0 reported and used to calculate *d*.

*A* Age, *C* Cues, *ER* Eye of regard, *H* Handedness, *HU* Hand used to bisect, *LL* line length, *S*_*1*_ Sex (separate data for males and females), *S*_*2*_ Sex (Percentage male), *SA* Salience, *SD* Scan direction, *SP* spatial position

**Table 2:** Study characteristics of studies using the landmark task

	Study	Mean Age	N	Task	Recruited as a clinical control group?	Effect size (Cohen’s d)	Moderator analysis	Additional information
1	Benwell et al. (2014)	68.5	20	LM	N	0.15	A, H, LL, S_2_	Raw data obtained from authors. Long (243mm = 19.69°) and short (24.3mm = 1.99°) lines used in line length analysis.
2	Harvey et al. (2000)	71.0	18	LM	N	-0.26	A, C, H, S_2_	T-statistic vs 0 reported for left cue visible and right cue visible conditions. *d* calculated separately for each, then averaged.
3	Learmonth et al. (2017)	68.8	19	LM	N	0.03	A, H, LL, S_1_, S2	Raw data obtained from authors. N = 1 excluded from analysis. Long (208.9mm = 14.88°) and short (20.7mm = 1.49°) lines used in line length analysis.
4	Learmonth et al. (2018)	70.4	39	LM	N	-0.24	A, H, S_1_, S_2_	Raw data obtained from authors.
5	Schmitz and Peigneux (2011)	69.4	19	LM	N	0.45	A, H, S_2_	T-statistic against chance (i.e., 50% “left longer”) reported and used to calculate *d*.
6	Zeller and Hullin (2018)	75.0	59	LM	N	0.54	A, H	Cohen’s d reported against chance (i.e., 50% “left end closer”).

*A* Age, *C* Cues, *H* Handedness, *LL* line length, *S*_*1*_ Sex (separate data for males and females), *S*_*2*_ Sex (Percentage male)

### Overall Effect Estimates

#### Line Bisection Task:

**Fig. 2:**
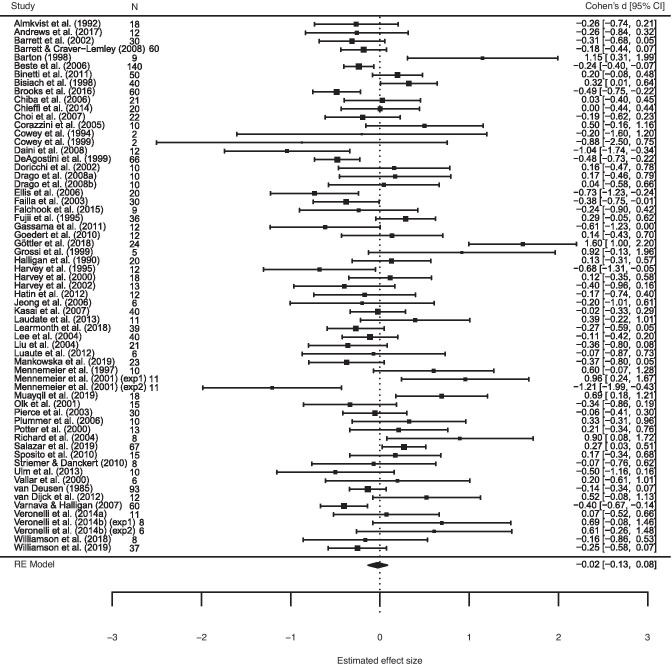
Mean effect size (Cohen’s d) and 95% confidence intervals for the 63 datasets included in the line bisection meta-analysis

**Fig. 3:**
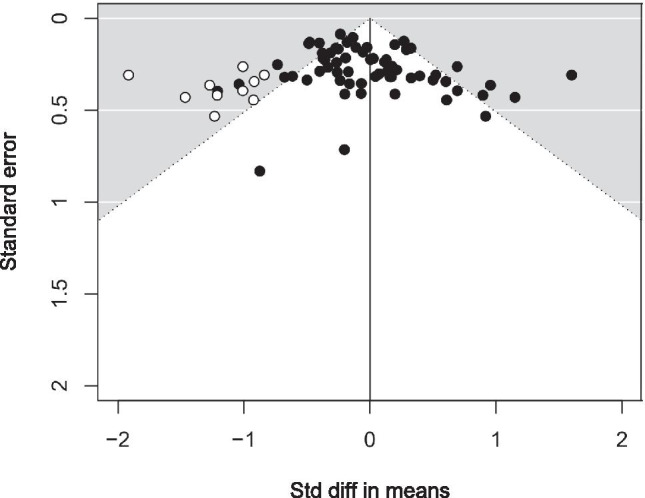
Funnel plot of standard errors by standard difference in means. The white circles represent the 10 imputed studies to the left of centre

A total of *k*_*d*_ = 63 datasets (from *k*_*t*_ = 61 studies) were included in the analysis, totalling *n*_*t*_ = 1480 individuals. A random effects model was employed, which gave a weighted average of the effect sizes across all datasets of *d* = -.02, and 95% confidence interval (95% CI) of -.13, .008, with no statistical evidence of a bias to either side of space, *Z* = -.44, *p* = .66 (Fig 2). The Egger’s *t*-test, revealed evidence of small study publication bias, *t* (61) = 2.13, *p* = .04, as did the visual inspection of the funnel plot (see Fig 3). According to the trim and fill test for random effects, 10 studies will need to be imputed to the left of the mean, corresponding to a smaller *d,* in order for the funnel plot to be symmetrical, in which case the effect size would be *d* = -.16, 95% CI = -.32, .01.

#### Landmark Task:

**Fig. 4:**
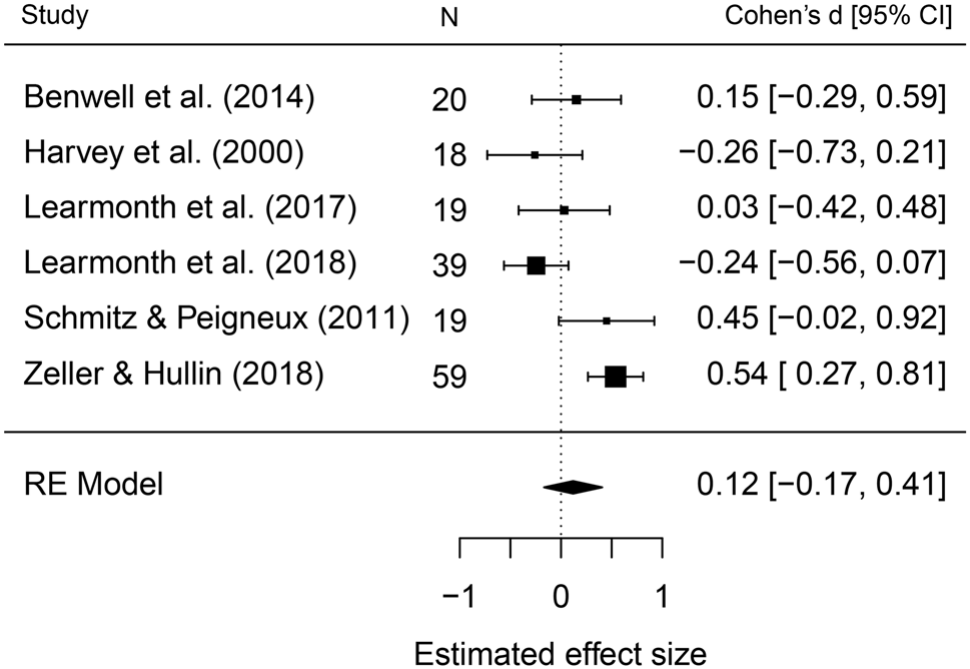
Mean effect size (Cohen’s d) and 95% confidence intervals for the 6 datasets included in the landmark task meta-analysis

A total of *k*_*d*_ = 6 datasets (from *k*_*t*_ = 6 studies) were included in the analysis, totalling *n* = 174 individuals. A random effects model gave a weighted average of the effect sizes across all datasets of *d* = .12, 95% CI = -.17, .41, *Z* = .82, *p* = .42 (Fig 4). In other words, we did not find evidence of a bias in the landmark task. Due to the small number of studies included in this analysis, the findings must be interpreted with caution. Moreover, no publication bias analysis or moderator variables analysis was justified.

### Comparison Between the Line Bisection and the Landmark Task

The 63 datasets that used the line bisection task were compared to the 6 datasets that used the landmark task. Of note, two of the studies (Harvey et al., 2000; Learmonth et al., 2018) administered the task to the same participants, therefore not all data points were independent. No statistical evidence of a difference between the two tasks was found, *Q* (1) = .85, *p* = .36. Again, due to the small number of studies that used the landmark task, caution is necessary when interpreting findings.

### Analysis of Moderator Variables: Line Bisection Task

The moderate inconsistency within the line bisection task datasets led us to test the possible moderating effect of age, line length, sex, the spatial position of the line, the presence of right of left lateralized cues and the control group status of the participants. Only the datasets that reported the information needed were included.

**Age:** The mean age of participants across all 63 datasets was 69.2 years. Meta-regression of the mean age of the participants in each dataset did not provide statistical evidence for a linear trend in pseudoneglect bias, *Q* (1) = 2.83, *p* = .09, *R*^2^ = 3.63%. Thirty-four datasets reported that all participants were at least 50 years old, and 29 datasets did not report the minimum age of their participants. When including only the studies where the minimum age was known to be ≥50, a random effects model identified a small leftward bias of *d* = -.16, 95% CI = -.26, -.05*, p* = .003. No bias was identified for the studies where the minimum age was not reported, *d* = .11, 95% CI = -.09, .32, *p* = .28. There was moderate heterogeneity for studies where participants were aged ≥50, *Q* (33) = 66.08, *p* = .0005, *I*^*2*^ = 50.23% and high heterogeneity where participants’ minimum age was not reported, *Q* (28) = 92.4, *p* < .0001, *I*^*2*^ = 76.95%. The two random models were statistically different to one another (*Z* = -23, *p* = .022). In an exploratory follow-up analysis of this effect, an interaction was identified between two factors: where the minimum age was known or unknown, and the control group status of the participants (where they were recruited as part of a stand-alone older group or as a clinical control group; see 6) Control group status below), *Q* (3) = 8.93, *p* = .03, *R*^2^ = 29.03%. Paired comparisons identified a difference between studies where participants were all ≥50 and the clinical controls, *Q* (1) = 5.14, *p* = .023, and between studies where the minimum age was unknown and the stand-alone older adult group, *Q* (1) = 6.2, *p* = .013.
**i) Sex:** Only 9 studies reported data separately for males and females (Barrett et al., 2002; Barrett & Craver-Lemley, 2008; Beste et al., 2006; De Agostini et al., 1999; Halligan et al., 1990; Learmonth et al., 2018; Muayqil et al., 2019; Pierce et al., 2003; Varnava & Halligan, 2007). When comparing the effect sizes for males and females, no evidence of a moderating effect was found, *Q* (1) = .66, *p* = .42, *R*^2^ = 0%. Specifically, the effect size was found to be *d* = -.06, *p* = .64, 95% CI = -.32, .19 for the male participants, with a small leftward bias of *d* = -.22, *p* = .04, 95% CI = -.42, -.01 for the female participants. Heterogeneity was further examined and revealed moderate heterogeneity within the datasets that represent male participants, *Q* (8) = 24.42, *p* = .002, *I*^*2*^ =67.67%, and moderate heterogeneity within the datasets that represent female participants, *Q* (8) = 17.7, *p* = .024, *I*^*2*^ = 54.3%. However, the small number of studies included does not allow for safe conclusions to be drawn.
  **ii) Percentage male:** Meta-regression of the percentage of male participants in each dataset (*n* = 42 datasets from 42 studies) did not provide statistical evidence of a linear trend in pseudoneglect bias, *Q* (1) = .25, *p* = .62, *R*^2^ = 0%.
**Line length:** Only 9 datasets (from 9 studies) reported data separately for the short and long line lengths (Binetti et al., 2011; Cowey et al., 1994; Ellis et al., 2006; Halligan et al., 1990; Kasai et al., 2016; Potter et al., 2000; Richard et al., 2004; Vallar et al., 2000; Varnava & Halligan, 2007). There was no statistical evidence of a moderating effect of line length, *Q* (1) = 1.64, *p* = .20, *R*^2^ = 4.48%. Specifically, the effect size was found to be *d* = .07, 95% CI = -.20, .35 when the lines were long and *d* = -.23, 95% CI = -.64, .18 when the lines were short. Heterogeneity was further examined and revealed high levels of heterogeneity within the studies that used long lines, *Q* (8) = 30.06, *p* = .0002, *I*^*2*^ = 69.88%, and high levels within the studies that used short lines, *Q* (8) = 41.88, *p* < .0001, *I*^*2*^ = 85.8%.
**Spatial position: **Only 9 datasets (from 8 studies) reported data separately for the position of the line (Beste et al., 2006; Ellis et al., 2006; Jeong et al., 2006; Learmonth et al., 2018; Mennemeier et al., 1997, 2001 (experiments 1 and 2); Potter et al., 2000; Williamson et al., 2018). No evidence of a moderating effect of line position was found, *Q* (1) = 1.10, *p* = .29, *R*^2^ = 0%. Specifically, the effect size was found to be *d* = -.28, 95% CI = -.58, .01 when the line was positioned to the left and *d* = .03, 95% CI = -.51, .57 when the line was positioned to the right. Heterogeneity was further examined and revealed moderate heterogeneity within the datasets where the line was positioned to the left, *Q* (8) = 20.71, *p* = .008, *I*^*2*^ = 70.57% and high heterogeneity within the datasets where the line was positioned to the right, *Q* (8) = 45.0, *p* < .0001, *I*^*2*^ = 91.27%. Similarly, the small number of studies included does not allow for safe conclusions to be drawn.
**Cues:** Six studies reported data separately for left- or right-sided cues (Drago et al., 2008a; 2008b; Falchook et al., 2015; Harvey et al., 2000; Mennemeier et al., 1997; Williamson et al., 2018). There was no statistical evidence of a moderating effect of cue direction, *Q* (1) = .054, *p* = .46, *R*^2^ = 0%. Specifically, the effect size was found to be *d* = -.09, 95% CI = -.36, .17 when there were left-sided cues and *d* = -.10, 95% CI = -.33, .54 when there were right lateralized cues. Heterogeneity was further examined and revealed low levels of heterogeneity within the studies that provided left lateralized cues, *Q* (5) = 4.76, *p* = .45, *I*^*2*^ = 11.41% and moderate levels of heterogeneity within the studies that provided right lateralized cues, *Q* (5) = 14.09, *p* = .02, *I*^*2*^ = 63.68%.
**Control group status:** There was marginal statistical evidence of a moderating effect of the participants’ control group status (i.e., whether they were recruited as control participants for a clinical study or they were investigated as a stand-alone older adult group), *Q* (1) = 3.97, *p* = .046, *R*^2^ = 10.99%. Specifically, the effect size was found to be *d* = .006, 95% CI = -.10, .23 when participants were controls to a clinical group (43 studies) and *d* = -.17, 95% CI = -.28, -.05 when they were healthy volunteers (20 studies). Heterogeneity was further examined and revealed moderate levels of heterogeneity within the studies that used controls to clinical groups, *Q* (42) = 120.12, *p* < .0001, *I*^*2*^ = 69.55%, and moderate heterogeneity within the studies that used healthy volunteers, *Q* (19) = 44.64, *p* = .0008, *I*^*2*^ = 59.67%.

## Discussion

We performed two meta-analyses to assess the evidence for a group-level lateralized spatial attention bias in adults aged 50 years old or older. Based on the prior meta-analysis of Jewell and McCourt (2000) and other studies (Benwell et al., 2014; Fujii et al., 1995; Fukatsu et al., 1990; Schmitz & Peigneux, 2011; Stam & Bakker, 1990), we hypothesised that older adults would exhibit a rightward spatial bias on the line bisection and landmark tasks (i.e., a mirrored bias relative to the leftward pseudoneglect that is typically observed in young adults). Separate meta-analyses were performed for the two spatial attention tasks and we also assessed the influence of six moderator variables: age, sex, line length, the spatial position of the line, the presence of left or right lateralised cues, and the control group status of the participants. A total of 69 datasets were included in the meta-analysis (63 in the line bisection analysis and 6 in the landmark analysis), comprising 1654 participants.

Overall, in contrast to our hypothesis, we identified no evidence of a lateralised bias in older adults for the line bisection task (*d* = -.09, 95% CI= -.19, .06). However, we identified a leftward line bisection bias for studies where all individual participants were known to be aged ≥50 (*d* = -.16, 95% CI = -.26, -.05, *p* = .003). There was no bias when the minimum age of participants was not reported (*d* = .11, 95% CI = -.09, .32, *p* = .28). This was a surprising finding because the inclusion of slightly younger participants would be expected to increase, rather than eliminate, a group-level leftward bias. Further exploration of the data suggested that this age effect was moderated by different participant characteristics across the two subgroups. The majority of studies in which the minimum age was not reported (and in which some participants *may* have been aged under 50) had also disproportionately been recruited as clinical controls (*n* = 24/29). This is important because clinical controls were found to have no bias to either side of space (*d* = .006, 95% CI = -.10, .23). Conversely, around half (*n* = 16/34) of studies in which all of the participants were aged ≥50 had been recruited as a stand-alone older adult group, a group in which we found a clear leftward bias (*d* = -.17, 95% CI = -.28, -.05). This suggests that the leftward bias that we observed in the older group was not eliminated by the inclusion of potentially younger participants but that there may be additional, and as yet unquantified, characteristics of clinical control participants that are different to the typical older adult population. This issue is discussed further below.

Moderator analysis for the line bisection task indicated that spatial biases were not moderated by mean age, sex, line length, line position, or the presence of lateralised cues. The Egger’s *t*-test identified a small study bias, a form of publication bias, in the line bisection literature with a small bias in favour of reporting rightward bisection errors. However, the corrected model indicated that the true effect size is likely to lie somewhere between *d* = -.32 and .01 which does retain the possibility of an eliminated bias after publication bias is accounted for. Taken together, we therefore tentatively estimate that spatial attention asymmetry in adults aged over 50 years old is likely to be slightly left of centre, similar to the pseudoneglect bias observed in young adults (Bowers & Heilman, 1980). Finally, we found no evidence of a lateralised spatial bias for the landmark task, although the number of studies included in this analysis was small, thus this finding should be treated with caution (*n* = 6, *d* = 0.12, 95% CI = -0.17, 0.44).

The observation of eliminated or reversed (i.e., rightward) spatial biases in seniors is often interpreted as evidence for either an asymmetry reduction, for example the HAROLD model of aging (Cabeza, 2002; Cabeza et al., 1997) or an advanced right hemisphere aging within the spatial attention networks (Goldstein & Shelly, 1981). These models of aging were principally developed from neuroimaging evidence of cortical reorganisation during working memory tasks and have been co-opted into the lateralised spatial attention domain to explain potential age-related changes in spatial biases. We found no evidence of a rightward bias for either task which might indicate a reversed asymmetry in this age group. If such behavioural measures of spatial bias are indeed reflective of underlying cortical asymmetries, then our results hint that the right hemisphere may retain dominance for spatial attention relative to the left hemisphere into older age (see Brooks et al., 2016). However, it is important to note that our conclusions must remain limited to the line bisection and landmark tasks, given that spatial biases tend not to be consistent in either their direction or magnitude when assessed using different measures (Learmonth et al., 2015a, 2015b; Märker et al., 2019; Mitchell et al., 2020; Nicholls et al., 1999).

Spatial attention biases are both stimulus- and state-dependent. The magnitude and direction of such biases are known to vary, for example in response to the presentation of different types of tasks (Brooks et al., 2016; Learmonth et al., 2015a; Luh et al., 1991; Nicholls et al., 1999), within-task features, such as the length of the bisected line (Benwell et al., 2014; Learmonth et al., 2017; Mennemeier et al., 2001), the viewing distance (Longo & Lourenco, 2006), and participant characteristics, for example their handedness (De Agostini et al., 1999; Failla et al., 2003) and alertness (Bellgrove et al., 2004; Dufour et al., 2007; Manly et al., 2005). In contrast to computerised versions of the line bisection, landmark, greyscales and gratingscales tasks, paper and pencil versions of the line bisection task typically require that a motor action is performed, usually using one hand. It is therefore not trivial to dissociate the contribution of pure visuospatial attention asymmetries here from the asymmetry derived from this unilateral action. However, the majority of studies included in these meta-analyses involved participants using only their right hand to bisect the line, which would be expected to preferentially activate the left motor cortex and give rise to a weaker leftward bias than bisection performed using the left hand (De Agostini et al., 1999; Failla et al., 2003; Ochando & Zago, 2018).

The meta-analysis of the line bisection task did not identify an influence of five of the six moderator variables that we included, namely age, sex, line length, the spatial position and the presence of left or right lateralised cues. In addition, we were unable to assess the effects of 7 variables that we had intended to analyse, either due to studies failing to report the necessary information (e.g., the percentage of left- and right-handers), or having identified too few studies that had experimentally manipulated the factor of interest in our target age group (e.g., the modality of line presentation, line salience, eye of regard, gaze direction, scanning direction, and body orientation). It is important to emphasise that the power of meta-analyses with respect to moderator variables relies on the number of studies that are included, rather than the number of participants. As such, these variables may yet be found to drive rightward shifts of spatial bias in older people, but we were unable to answer this question here. We recommend that further studies be performed to specifically assess the influence of these moderator variables, for studies to report their data by gender and handedness and also provide raw data to inform future meta-analyses.

The presence of a moderating effect of control group status was a surprising finding. We identified a small leftward bias in studies which had recruited a stand-alone group of older adults (e.g., in a comparison of young *vs* older adults) but no bias in studies which had recruited older people as age-matched control participants in a clinical study. It is not immediately apparent what the reason for this difference might be, although there may be differences in the recruitment strategies adopted across the two groups. It may be that older people who are willing and able to attend university laboratories to take part in research studies are less representative of their peers across a range of characteristics (e.g., they may be healthier, more active, and better educated; Ganguli et al., 1998; Golomb et al., 2012). If these characteristics are protective against, or negatively correlated with, age-related cortical reorganisation, we may not expect to see shifts in spatial bias in such a highly performing sample of older people. Clinicians should likewise aim to ensure that control participants are representative of the wider population when enrolling participants into clinical studies. In line with this, we recommend that older recruits be screened for cognitive decline (e.g., using the Montreal Cognitive Assessment; Nasreddine et al., 2005) and visual loss (see Learmonth et al., 2017) prior to taking part in spatial attention experiments. Given the small magnitude of asymmetries that are typically observed on the line bisection and landmark tasks, any pre-existing visual or cognitive impairments in this age group could reduce the precision of the bisection judgements that are made.

In addition to these screening measures, we recommend performing and pre-registering sample size calculations prior to commencing each study. Although we found that a minority of studies recruited a relatively large number of participants (*n* = 60 Barrett & Craver-Lemley, (2008); *n* = 140 Beste et al., (2006); *n* = 66 De Agostini et al., (1999); *n* = 67 Salazar et al., 2019; *n* = 93 Van Deusen, (1983); *n =* 60 Varnava & Halligan, (2007); *n =* 59 Zeller & Hullin, (2018), the median sample size across all studies included in the analysis was only 15 participants. The standard statistical analyses that are performed in spatial bias experiments tend to be reliant upon a comparison of group-level means (e.g., one sample *t*-tests and analysis of variance). A high level of inter-individual variability of spatial bias is often observed in both young and older adults (Benwell et al., 2013; Learmonth et al., 2015a, 2015b; Märker et al., 2019; McCourt, 2001; Newman et al., 2014; Szczepanski & Kastner, 2013), meaning that a large sample size is an important factor in obtaining a robust estimation of the mean bias. Under-recruitment is likely to result in studies that are susceptible to both false positive and false negative findings (Minarik et al., 2016) that may lead to a skewed estimation of spatial biases in the general population.

We acknowledge that there are a number of potential limitations to this meta-analysis. Although we found some evidence of a maintained directional spatial bias in older adults, we did not determine whether this bias is specifically reduced in magnitude compared to young adults. A meta-analysis to compare effect sizes obtained in the two age groups should now be undertaken to specifically address this question. Secondly, based on the methodology of Jewell and McCourt (2000), we included studies where the mean age was at least 50 years old. This is perhaps slightly young compared to many studies of age-related changes in spatial bias, which often use 60 years old as the lower bound, however, the mean age across all studies here was 69.3 years. Because we also failed to find a moderating influence of mean age in the line bisection analysis, we conclude that spatial bias is likely to be stable in the over-50s population, although we must also acknowledge that a considerable number of studies (*n* = 18) were excluded due to a lack of sufficient data available with which to derive an effect size.

Another potentially limiting factor is that we constrained our analysis to only two of the most commonly used measures of spatial attention bias, namely, the landmark and line bisection tasks. Our own prior research has highlighted that the magnitude and direction of spatial biases often do not correlate across different tasks in young adults (Brooks et al., 2016; Learmonth et al., 2018; Learmonth et al., 2015a; Luh, 1995; Nicholls et al., 1999) or in older adults (Märker et al., 2019). It is possible that alternative tasks, perhaps those involving luminance (e.g., greyscales; Mattingley et al., 1994) or spatial frequency judgements (e.g., gratingscales; Niemeier et al., 2007) are more effective in detecting subtle age-related changes in spatial bias compared to the line bisection and landmark tasks. However, a systematic review by Friedrich et al. (2016) suggests that this may not be the case, with the oldest adults tested (aged 80-89 years) exhibiting a stronger leftward bias on the greyscales task than young adults, aged 18-29. At present there are few studies available which detail older adult performance on these alternative tasks. Again, we recommend that additional studies be undertaken using these measures in order to adequately address this question.

### Conclusions

In conclusion, we identified no overall spatial attention bias to either side of space, as indexed by the line bisection and landmark tasks, for studies where the mean page of participants was ≥50 years old. However, there was a clear leftward line bisection bias for studies where all of the individual participants were known to be aged at least 50 and no bias for studies where the minimum age of participants was not reported. Secondly, a leftward line bisection bias was observed in studies where participants were recruited as a stand-alone older group, but there was no bias in studies where participants were clinical controls. No other moderating effects were identified and there was evidence of a small publication bias in favour of reporting rightward line bisection biases. These results suggest that older adults are likely to maintain a small leftward line bisection bias similar to young adults, but more studies are recommended in order to assess the potentially moderating influence of stimulus- and state-dependent factors.

## References

[CR1] Almkvist O, Basun H, Wahlund LO, Andersson Lundman G, Bäckman L (1992). White-matter hyperintensity and neuropsychological functions in dementia and healthy aging. Archives of Neurology.

[CR2] Andrade, K., Kas, A., Samri, D., Sarazin, M., Dubois, B., Habert, M.-O., & Bartolomeo, P. (2013). Visuospatial deficits and hemispheric perfusion asymmetries in posterior cortical atrophy. *Cortex*, *49*(4), 940–947. 10.1016/j.cortex.2012.03.010 10.1016/j.cortex.2012.03.010 22513341

[CR3] Andrews B, D’Avossa G, Sapir A (2017). Aging changes 3D perception: Evidence for hemispheric rebalancing of lateralized processes. Neuropsychologia.

[CR4] Arduino LS, Veronelli L, Cai L, Xue S, Corbo M, Zhang Y (2016). Pseudoneglect in sentence bisection: a comparison between Italian and Chinese. Journal of Cognitive Psychology.

[CR5] Baheux K, Yoshizawa M, Yoshida Y (2007). Simulating hemispatial neglect with virtual reality. Journal of Neuroengineering and Rehabilitation.

[CR6] Bailey MJ, Riddoch MJ, Crome P (2000). Evaluation of a test battery for hemineglect in elderly stroke patients for use by therapists in clinical practice. NeuroRehabilitation.

[CR7] Balconi M, Amenta S, Sozzi M, Cannatà AP, Pisani L (2013). Eye movement and online bisection task in unilateral patients with neglect: a new look to the “gradient effect”. Brain Injury.

[CR8] Barrett AM, Craver-Lemley CE (2008). Is it what you see, or how you say it? Spatial bias in young and aged subjects. Journal of the International Neuropsychological Society.

[CR9] Barrett AM, Kim M, Crucian GP, Heilman KM (2002). Spatial bias: Effects of early reading direction on Korean subjects. Neuropsychologia.

[CR10] *Barton, J. J. S., Behrmann, M., & Black, S. (1998). Ocular search during line bisection. The effects of hemi-neglect and hemianopia. *Brain*, *121*(6), 1117–1131. 10.1093/brain/121.6.1117 10.1093/brain/121.6.1117 9648547

[CR11] Bellgrove MA, Dockree PM, Aimola L, Robertson IH (2004). Attenuation of spatial attentional asymmetries with poor sustained attention. Neuroreport.

[CR12] Benwell CSY, Thut G, Grant A, Harvey M (2014). A rightward shift in the visuospatial attention vector with healthy aging. Frontiers in aging neuroscience.

[CR13] Benwell CSY, Thut G, Learmonth G, Harvey M (2013). Spatial attention: differential shifts in pseudoneglect direction with time-on-task and initial bias support the idea of observer subtypes. Neuropsychologia.

[CR14] Beste C, Hamm JP, Hausmann M (2006). Developmental changes in visual line bisection in women throughout adulthood. Developmental Neuropsychology.

[CR15] Binetti N, Aiello M, Merola S, Bruschini M, Lecce F, Macci E, Doricchi F (2011). Positive correlation in the bisection of long and short horizontal Oppel-Kundt illusory gradients: Implications for the interpretation of the “cross-over” effect in spatial neglect. Cortex.

[CR16] Bisiach E, Ricci R, Mòdona MN (1998). Visual Awareness and Anisometry of Space Representation in Unilateral Neglect: A Panoramic Investigation by Means of a Line Extension Task. Consciousness and Cognition.

[CR17] Bourne VJ, Gray DL (2009). Hormone exposure and functional lateralisation: Examining the contributions of prenatal and later life hormonal exposure. Psychoneuroendocrinology.

[CR18] Bowers D, Heilman KM (1980). Pseudoneglect: Effects of hemispace on a tactile line bisection task. Neuropsychologia.

[CR19] Brooks JL, Darling S, Malvaso C, Della Sala S (2016). Adult developmental trajectories of pseudoneglect in the tactile, visual and auditory modalities and the influence of starting position and stimulus length. Brain and Cognition.

[CR20] Brooks, J. L., Sala, S. Della, & Logie, R. H. (2011). Tactile rod bisection in the absence of visuo-spatial processing in children, mid-age and older adults. *Neuropsychologia*, *49*(12), 3392–3398. 10.1016/j.neuropsychologia.2011.08.015 10.1016/j.neuropsychologia.2011.08.015 21875610

[CR21] Cabeza R (2002). Hemispheric asymmetry reduction in older adults: the HAROLD model. Psychology and aging.

[CR22] Cabeza R, Anderson ND, Locantore JK, McIntosh AR (2002). Aging gracefully: Compensatory brain activity in high-performing older adults. NeuroImage.

[CR23] Cabeza R, Grady CL, Nyberg L, McIntosh AR, Tulving E, Kapur S (1997). Age-related differences in neural activity during memory encoding and retrieval: A positron emission tomography study.

[CR24] Chen P, Goedert KM (2012). Clock drawing in spatial neglect: A comprehensive analysis of clock perimeter, placement, and accuracy. Journal of Neuropsychology.

[CR25] Chiba Y, Yamaguchi A, Eto F (2006). Assessment of sensory neglect: A study using moving images. Neuropsychological Rehabilitation.

[CR26] Chieffi S, Iavarone A, Iaccarino L, La Marra M, Messina G, De Luca V, Monda M (2014). Age-related differences in distractor interference on line bisection. Experimental Brain Research.

[CR27] Choi KM, Lee BH, Lee SC, Ku BD, Kim EJ, Suh MK (2007). Influence of moving background on line bisection performance in the normal elderly versus patients with hemispatial neglect. American Journal of Physical Medicine and Rehabilitation.

[CR28] Çiçek M, Deouell LY, Knight RT (2009). Brain activity during landmark and line bisection tasks. Frontiers in human neuroscience.

[CR29] Corazzini LL, Geminiani G, Stucchi N, Gindri P, Cremasco L (2005). Visual acceleration and spatial distortion in right brain-damaged patients. Experimental Brain Research.

[CR30] Costantini M, Frassinetti F, Maini M, Ambrosini E, Gallese V, Sinigaglia C (2014). When a laser pen becomes a stick: remapping of space by tool-use observation in hemispatial neglect. Experimental Brain Research.

[CR31] Cowey A, Small M, Ellis S (1994). Left visuo-spatial neglect can be worse in far than in near space. Neuropsychologia.

[CR32] Cowey A, Small M, Ellis S (1998). No abrupt change in visual hemineglect from near to far space. Neuropsychologia.

[CR33] Daini R, Arduino LS, Di Menza D, Vallar G, Silveri MC (2008). Line bisection and cerebellar damage. Cognitive and Behavioral Neurology.

[CR34] De Agostini M, Curt F, Tzortzis C, Dellatolas G (1999). Comparing left and right hand in line bisection at different ages. Developmental Neuropsychology.

[CR35] Dolcos F, Rice HJ, Cabeza R (2002). Hemispheric asymmetry and aging: right hemisphere decline or asymmetry reduction. Neuroscience & Biobehavioral Reviews.

[CR36] *Doricchi, F., Onida, A., & Guariglia, P. (2002). Horizontal space misrepresentation in unilateral brain damage II. Eye-head centered modulation of visual misrepresentation in hemianopia without neglect. *Neuropsychologia*, *40*(8), 1118–1128. 10.1016/S0028-3932(02)00011-8 10.1016/s0028-3932(02)00011-8 11931916

[CR37] Drago V, Foster PS, Ferri R, Arico D, Lanuzza B, Heilman KM (2008). Distractibility and Alzheimer disease: The “neglected” phenomenon. Journal of Alzheimer’s Disease.

[CR38] Drago V, Foster PS, Skidmore FM, Kluger B, Antoniello D, Heilman KM (2008). Attentional grasp in Parkinson disease. Cognitive and Behavioral Neurology.

[CR39] Dufour A, Touzalin P, Candas V (2007). Time-on-task effect in pseudoneglect. Experimental Brain Research.

[CR40] Duval S, Tweedie R (2000). Trim and fill: A simple funnel-plot-based method of testing and adjusting for publication bias in meta-analysis. Biometrics.

[CR41] Ebersbach, G., Trottenberg, T., Hättig, H., Schelosky, L., Schrag, A., & Poewe, W. (1996). Directional bias of initial visual exploration. A symptom of neglect in Parkinson’s disease. *Brain*, *119*(1), 79–87. 10.1093/brain/119.1.79 10.1093/brain/119.1.79 8624696

[CR42] Ellis AW, Jordan JL, Sullivan CA (2006). Unilateral neglect is not unilateral: Evidence for additional neglect of extreme right space. Cortex.

[CR43] Failla CV, Sheppard DM, Bradshaw JL (2003). Age and responding-hand related changes in performance of neurologically normal subjects on the line-bisection and chimeric-faces tasks. Brain and Cognition.

[CR44] Falchook AD, Salazar L, Neal D, Kesayan T, Williamson JB, Malaty IA (2015). Global attentional neglect of segmented lines in Parkinson’s disease. Neurocase.

[CR45] Fink GR, Marshall JC, Weiss PH, Zilles K (2001). The Neural Basis of Vertical and Horizontal Line Bisection Judgments : An fMRI Study of Normal Volunteers. NeuroImage.

[CR46] Fisher, Z., Tipton, E., & Zhipeng, H. (2017). Robumeta: Robust variance meta-regression. R package version 2.0.

[CR47] Foxe JJ, McCourt ME, Javitt DC (2003). Right hemisphere control of visuospatial attention: line-bisection judgments evaluated with high-density electrical mapping and source analysis☆. NeuroImage.

[CR48] Friedrich TE, Hunter PV, Elias LJ (2016). Developmental trajectory of pseudoneglect in adults using the greyscales task. Developmental Psychology.

[CR49] Friedrich TE, Hunter PV, Elias LJ (2018). The trajectory of pseudoneglect in adults: A systematic review. Neuropsychology Review.

[CR50] Fujii T, Fukatsu R, Yamadori A, Kimura I (1995). Effect of age on the line bisection test. Journal of Clinical and Experimental Neuropsychology.

[CR51] Fukatsu R, Fujii T, Kimura I, Saso S, Kogure K (1990). Effects of hand and spatial conditions on visual line bisection. The Tohoku Journal of Experimental Medicine.

[CR52] Ganguli, M., Lytle, M. E., Reynolds, M. D., & Dodge, H. H. (1998). Random versus volunteer selection for a community-based study. *The Journals of Gerontology. Series A: Biological Sciences and Medical Sciences*, *53*(1), M39-46. 10.1093/gerona/53a.1.m39 10.1093/gerona/53a.1.m39 9467432

[CR53] Gassama S, Deplancke A, Saj A, Honoré J, Rousseaux M (2011). Do supine position and deprivation of visual environment influence spatial neglect?. Journal of Neurology.

[CR54] Goedert KM, Leblanc A, Tsai SW, Barrett AM (2010). Asymmetrical effects of adaptation to left- and right-Shifting prisms depends on pPre-existing attentional biases. Journal of the International Neuropsychological Society.

[CR55] Goldstein G, Shelly C (1981). Does the right hemisphere age more rapidly than the left?. Journal of Clinical Neuropsychology.

[CR56] Golomb BA, Chan VT, Evans MA, Koperski S, White HL, Criqui MH (2012). The older the better: are elderly study participants more non-representative? A cross-sectional analysis of clinical trial and observational study samples. BMJ open.

[CR57] *Göttler, J., Kaczmarz, S., Nuttall, R., Griese, V., Napiórkowski N., Kallmayer, M. et al. (2018). The stronger one-sided relative hypoperfusion, the more pronounced ipsilateral spatial attentional bias in patients with asymptomatic carotid stenosis. *Journal of Cerebral Blood Flow and Metabolism, 1*(14). 10.1177/0271678X18815790 10.1177/0271678X18815790 PMC7370612 30480463

[CR58] Grossi D, Lepore M, Esposito A, Napolitano A, Serino M, Trojano L (1999). Neglect-associated constructional disorders: A paradoxical phenomenon?. Neuropsychologia.

[CR59] Gutiérrez Pérez C, Sävborg M, Påhlman U, Cederfeldt M, Knopp E, Nordlund A (2011). High frequency of cognitive dysfunction before stroke among older people. International Journal of Geriatric Psychiatry.

[CR60] Halligan PW, Manning L, Marshall JC (1990). Individual variation in line bisection: A study of four patients with right hemisphere damage and normal controls. Neuropsychologia.

[CR61] Harvey M, Krämer-McCaffery T, Dow L, Murphy PJS, Gilchrist ID (2002). Categorisation of “perceptual” and “premotor” neglect patients across different tasks: Is there strong evidence for a dichotomy?. Neuropsychologia.

[CR62] Harvey M, Milner AD, Roberts RC (1995). Differential Effects of Line Length on Bisection Judgements in Hemispatial Neglect. Cortex.

[CR63] Harvey M, Milner AD, Roberts RC (1995). An investigation of hemispatial neglect using the landmark task. Brain and Cognition.

[CR64] Harvey M, Pool TD, Roberson MJ, Olk B (2000). Effects of visible and invisible cueing procedures on perceptual judgments in young and elderly subjects. Neuropsychologia.

[CR65] Hatin B, Sykes Tottenham L, Oriet C (2012). The relationship between collisions and pseudoneglect: Is it right?. Cortex.

[CR66] Higgins JPT, Thompson SG, Deeks JJ, Altman DG (2003). Measuring inconsistency in meta-analyses. BMJ.

[CR67] Jeong Y, Tsao JW, Efros DB, Heilman KM (2006). Callosal neglect in hydrocephalus. Neurocase.

[CR68] Jewell G, McCourt ME (2000). Pseudoneglect: a review and meta-analysis of performance factors in line bisection tasks. Neuropsychologia.

[CR69] Kasai M, Ishizaki J, Meguro K (2016). Alzheimer’s patients do not show left unilateral spatial neglect but exhibit peripheral inattention and simplification. Dementia & Neuropsychologia.

[CR70] Kim EJ, Kwon H, Lee BH, Kim GH, Seo SW, Na DL (2011). Attentional distractibility induced by optokinetic stimulation in mild cognitive impairment. Alzheimer Disease and Associated Disorders.

[CR71] Laudate TM, Neargarder S, Cronin-Golomb A (2013). Line bisection in Parkinson’s disease: Investigation of contributions of visual field, retinal vision, and scanning patterns to visuospatial function. Behavioral Neuroscience.

[CR72] Learmonth G, Benwell CSY, Thut G, Harvey M (2017). Age-related reduction of hemispheric lateralisation for spatial attention: An EEG study. NeuroImage.

[CR73] Learmonth G, Gallagher A, Gibson J, Thut G, Harvey M (2015). Intra- and inter-task reliability of spatial attention measures in pseudoneglect. PLoS One.

[CR74] Learmonth G, Märker G, McBride N, Pellinen P, Harvey M (2018). Right-lateralised lane keeping in young and older British drivers. PLoS One.

[CR75] Learmonth G, Thut G, Benwell CSY, Harvey M (2015). The implications of state-dependent tDCS effects in aging: Behavioural response is determined by baseline performance. Neuropsychologia.

[CR76] Lee AC, Harris JP, Atkinson EA, Fowler MS (2001). Evidence from a line bisection task for visuospatial neglect in left hemiparkinson’s disease. Vision Research.

[CR77] Lee AC, Harris JP, Atkinson EA, Nithi K, Fowler MS (2002). Dopamine and the representation of the upper visual field: evidence from vertical bisection errors in unilateral Parkinson’s disease. Neuropsychologia.

[CR78] Lee BH, Kim M, Kang SJ, Park KC, Kim E-JJ, Adair JC, Na DL (2004). Pseudoneglect in solid-line versus character-line bisection tasks: A test for attention dominance theory. Cognitive and Behavioral Neurology.

[CR79] Lee Byung Hwa, Kang E, Cho SS, Kim EJ, Seo SW, Kim GM (2010). Neural correlates of hemispatial neglect: a voxel-based SPECT study. Cerebrovascular diseases.

[CR80] Levy J, Trevarthen C, Sperry R (1972). Perception of bilateral chimeric figures following hemispheric disconnexion. Brain.

[CR81] Liu CJ, McDowd J, Lin KC (2004). Visuospatial inattention and daily life performance in people with Alzheimer’s disease. American Journal of Occupational Therapy.

[CR82] Longo MR, Lourenco SF (2006). On the nature of near space: Effects of tool use and the transition to far space. Neuropsychologia.

[CR83] Luauté J, Jacquin-Courtois S, O’Shea J, Christophe L, Rode G, Boisson D, Rossetti Y (2012). Left-deviating prism adaptation in left neglect patient: Reflexions on a negative result. Neural Plasticity.

[CR84] Luh KE (1995). Line bisection and perceptual asymmetries in normal individuals: What you see is not what you get. Neuropsychology.

[CR85] Luh KE, Rueckert LM, Levy J (1991). Perceptual asymmetries for free viewing of several types of chimeric stimuli. Brain and Cognition.

[CR86] Luvizutto, G. J., Fogaroli, M. O., Theotonio, R. M., Moura Neto, E. D., Nunes, H. R. D. C., & Bazan, R. (2020). Norm scores of cancelation and bisection tests for unilateral spatial neglect: data from a Brazilian population. *Clinics, 75*. 10.6061/clinics/2019/e1468 10.6061/clinics/2019/e1468 PMC7196728 32401966

[CR87] Mańkowska A, Heilman KM, Williamson JB, Michałowski J, Harciarek M (2019). Age-related changes in the allocation of spatially directed focal attention. Aging, Neuropsychology, and Cognition.

[CR88] Manly T, Dobler VB, Dodds CM, George MA (2005). Rightward shift in spatial awareness with declining alertness. Neuropsychologia.

[CR89] Märker G, Learmonth G, Thut G, Harvey M (2019). Intra- and inter-task reliability of spatial attention measures in healthy older adults. PLoS One.

[CR90] Mattingley, J. B., Bradshaw, J. L., Nettleton, N. C., & Bradshaw, J. A. (1994). Can task specific perceptual bias be distinguished from unilateral neglect? *Neuropsychologia*, *32*(7), 805–817. 10.1016/0028-3932(94)90019-1 10.1016/0028-3932(94)90019-1 7936164

[CR91] McCourt ME (2001). Performance consistency of normal observers in forced-choice tachistoscopic visual line bisection. Neuropsychologia.

[CR92] McCourt ME, Garlinghouse M, Reuter-Lorenz PA (2005). Unilateral visual cueing and asymmetric line geometry share a common attentional origin in the modulation of pseudoneglect. Cortex.

[CR93] McCourt, M. E., Garlinghouse, M., & Slater, J. (2000). Centripetal versus centrifugal bias in visual line bisection: focusing attention on two hypotheses. *Frontiers In Bioscience*, *5*(D), 58–71. 10.2741/a496 10.2741/a496 10702377

[CR94] McCourt ME, Jewell G (1999). Visuospatial attention in line bisection: stimulus modulation of pseudoneglect. Neuropsychologia.

[CR95] Mennemeier M, Rapcsak SZ, Pierce C, Vezey E (2001). Crossover by line length and spatial location. Brain and Cognition.

[CR96] Mennemeier M, Vezey E, Chatterjee A, Rapcsak SZ, Heilman KM (1997). Contributions of the left and right cerebral hemispheres to line bisection. Neuropsychologia.

[CR97] Milner AD, Brechmann M, Pagliarini L (1992). To halve and to halve not: An analysis of line bisection judgements in normal subjects. Neuropsychologia.

[CR98] Minarik T, Berger B, Althaus L, Bader V, Biebl B, Brotzeller F (2016). The importance of sample size for reproducibility of tDCS effects. Frontiers in Human Neuroscience.

[CR99] Mitchell AG, Harris JM, Benstock SE, Ales JM (2020). The reliability of pseudoneglect is task dependent. Neuropsychologia.

[CR100] Moher, D., Liberati, A., Tetzlaff, J., Altman, D. G., & The PRISMA Group (2009). Preferred Reporting Items for Systematic Reviews and Meta-Analyses: The PRISMA Statement. Annals of Internal Medicine.

[CR101] *Muayqil, T. A., Al-Yousef, L. M., Al-Herbish, M. J., Al-Nafisah, M., Halawani, L. M., Al-Bader, S. S. et al. (2019). Culturally influenced performance on tasks of line bisection and symbol cancellation in Arabs. *Applied Neuropsychology:Adult, 1*(12). 10.1080/23279095.2019.1627359 10.1080/23279095.2019.1627359 31215237

[CR102] Nagamatsu LS, Carolan P, Liu-Ambrose TYL, Handy TC (2011). Age-related changes in the attentional control of visual cortex: A selective problem in the left visual hemifield. Neuropsychologia.

[CR103] Nagamatsu LS, Liu-Ambrose TYL, Carolan P, Handy TC (2009). Are impairments in visual-spatial attention a critical factor for increased falls risk in seniors?. An event-related potential study. Neuropsychologia.

[CR104] Nagamatsu LS, Munkacsy M, Liu-Ambrose TYL, Handy TC (2013). Altered visual-spatial attention to task-irrelevant information is associated with falls risk in older adults. Neuropsychologia.

[CR105] Nasreddine Z, Charbonneau S, Cummings JL (2005). The Montreal Cognitive Assessment, MoCA: a brief screening tool for mild cognitive impairment. Journal of the American Geriatrics Society.

[CR106] Newman DP, Loughnane GM, Abe R, Zoratti MTR, Martins ACP, van den Bogert PC (2014). Differential shift in spatial bias over time depends on observers׳ initial bias: Observer subtypes, or regression to the mean?. Neuropsychologia.

[CR107] Nicholls MER, Bradshaw JL, Mattingley JB (1999). Free-viewing perceptual asymmetries for the judgement of brightness, numerosity and size. Neuropsychologia.

[CR108] Niemeier M, Stojanoski BB, Greco AL (2007). Influences of time and spatial frequency on the perceptual bias: evidence for competition between hemispheres. Neuropsychologia.

[CR109] Ochando, A., & Zago, L. (2018). What are the contributions of handedness, sighting dominance, hand used to bisect, and visuospatial line processing to the behavioral line bisection bias? *Frontiers in Psychology*, *9*(SEP), 1–8. 10.3389/fpsyg.2018.01688 10.3389/fpsyg.2018.01688 PMC6143685 30258382

[CR110] Olk B, Harvey M, Dow L, Murphy PJS (2001). Illusion processing in hemispatial neglect. Neuropsychologia.

[CR111] Olk B, Wee J, Kingstone A (2004). The effect of hemispatial neglect on the perception of centre. Brain and Cognition.

[CR112] Pierce CA, Jewell G, Mennemeier M (2003). Are psychophysical functions derived from line bisection reliable?. Journal of the International Neuropsychological Society.

[CR113] Pizzamiglio L, Committeri G, Galati G, Patria F (2000). Psychophysical properties of line bisection and body midline perception in unilateral neglect. Cortex.

[CR114] Pizzamiglio L, Frasca R, Guariglia C, Incoccia C, Antonucci G (1990). Effect of optokinetic stimulation in patients with visual neglect. Cortex.

[CR115] Plummer P, Dunai J, Morris ME (2006). Understanding the effects of moving visual stimuli on unilateral neglect following stroke. Brain and Cognition.

[CR116] Potter J, Deighton T, Patel M, Fairhurst M, Guest R, Donnelly N (2000). Computer recording of standard tests of visual neglect in stroke patients. Clinical Rehabilitation.

[CR117] Reuter-Lorenz PA, Posner MI (1990). Components of neglect from right-hemisphere damage: An analysis of line bisection. Neuropsychologia.

[CR118] Richard C, Honoré J, Bernati T, Rousseaux M (2004). Straight-ahead pointing correlates with long-line bisection in neglect patients. Cortex.

[CR119] Rousseaux M, Bernati T, Saj A, Kozlowski O (2006). Ineffectiveness of prism adaptation on spatial neglect signs. Stroke.

[CR120] Salazar RD, Moon KLM, Neargarder S, Cronin-Golomb A (2019). Spatial judgment in Parkinson’s disease: Contributions of attentional and executive dysfunction. Behavioral Neuroscience.

[CR121] Schenkenberg T, Bradford DC, Ajax ET (1980). Line bisection and unilateral visual neglect in patients with neurologic impairment. Neurology.

[CR122] Schmitz RR, Peigneux P (2011). Age-related changes in visual pseudoneglect. Brain and cognition.

[CR123] Sposito AV, Bolognini N, Vallar G, Posteraro L, Maravita A (2010). The spatial encoding of body parts in patients with neglect and neurologically unimpaired participants. Neuropsychologia.

[CR124] Stam CJ, Bakker M (1990). The prevalence of neglect: superiority of neuro-psychological over clinical methods of estimation. Clinical Neurology and Neurosurgery.

[CR125] Striemer CL, Danckert J (2010). Dissociating perceptual and motor effects of prism adaptation in neglect. NeuroReport.

[CR126] Szczepanski SM, Kastner S (2013). Shifting attentional priorities: control of spatial attention through hemispheric competition. The Journal of Neuroscience.

[CR127] Ulm L, Wohlrapp D, Meinzer M, Steinicke R, Schatz A, Denzler P (2013). A circle-monitor for computerised assessment of visual neglect in peripersonal space. PLoS ONE.

[CR128] Vallar G, Daini R, Antonucci G (2000). Processing of illusion of length in spatial hemineglect: A study of line bisection. Neuropsychologia.

[CR129] Van Deusen J (1983). Normative data for ninety-three elderly persons on the Schenkenberg line bisection test. Physical and Occupational Therapy in Geriatrics.

[CR130] van Dijck J-P, Gevers W, Lafosse C, Fias W (2012). The Heterogeneous Nature of Number-Space Interactions. Frontiers in Human Neuroscience.

[CR131] Varnava A, Halligan PW (2007). Influence of age and sex on line bisection: A study of normal performance with implications for visuospatial neglect. Aging, Neuropsychology, and Cognition.

[CR132] Veronelli L, Guasti MT, Arduino LS, Vallar G (2014). Combining language and space: Sentence bisection in unilateral spatial neglect. Brain and Language.

[CR133] Veronelli L, Vallar G, Marinelli CV, Primativo S, Arduino LS (2014). Line and word bisection in right-brain-damaged patients with left spatial neglect. Experimental Brain Research.

[CR134] Viechtbauer W (2007). Accounting for heterogeneity via random-effects models and moderator analyses in meta-analysis. Zeitschrift für Psychologie/Journal of Psychology.

[CR135] Wang Q, Sonoda S, Hanamura M, Okazaki H, Saitoh E (2005). Line bisection and rebisection: The crossover effect of space location. Neurorehabilitation and Neural Repair.

[CR136] Williamson JB, Lamb DG, Burtis DB, Haque S, Zilli M, E., Kesayan, T.,  (2018). Right hemispatial ipsilesional neglect with chronic right hemisphere strokes. Journal of Clinical and Experimental Neuropsychology.

[CR137] *Williamson, J. B., Murphy, A., Lamb, D. G., Schwartz, Z., Szeles, D., Harciarek, M., et al. (2019). Improved accuracy on lateralized spatial judgments in healthy aging. *Journal of the International Neuropsychological Society**, **25*(10), 1044–1050. 10.1017/S1355617719000900 10.1017/S1355617719000900 31543083

[CR138] Zeller D, Hullin M (2018). Spatial attention and the malleability of bodily self in the elderly. Consciousness and Cognition.

